# Refractory linear IgA dermatosis in childhood: a successful response to rituximab^؆^

**DOI:** 10.1016/j.abd.2026.501308

**Published:** 2026-03-23

**Authors:** Pedro Sciammarella Wakisaka, Larissa Maria Cardoso Lima Rodrigues, Denise Miyamoto, Celina W. Maruta, Claudia Giuli Santi, Valeria Aoki

**Affiliations:** a Centro Universitário Faculdade de Medicina do ABC, Santo André, SP, Brazil; b Department of Dermatology, Hospital das Clínicas, Faculty of Medicine, Universidade de São Paulo, São Paulo, SP, Brazil

Dear editor,

Linear immunoglobulin A (IgA) bullous dermatosis (LABD) is an autoimmune blistering disease (AIBD) of idiopathic or drug-induced etiology. IgA autoantibodies may target full-length BP180 or its NC16A, LABD97, or LAD-1 regions, and occasionally BP230, leading to subepidermal blisters. Dapsone in monotherapy or association is the first-line treatment. Although treatment of LABD with dapsone is usually satisfactory, there are reports of refractory cases and severe adverse effects impairing disease control.^1^

A 4-year-old boy presented to the Dermatology clinic reporting the eruption of pruritic lesions over the last 8-months. He had no remarkable past medical history nor presented with systemic symptoms. Clinical examination showed tense vesicles with clear and hemorrhagic fluid, arranged in a “cluster of jewels” pattern around the nose, mouth, hard palate, trunk, pubic area, glutes, and limbs (Fig. 1). The skin lesions evolved with crusts and hyperpigmentation.

**Fig. 1:**
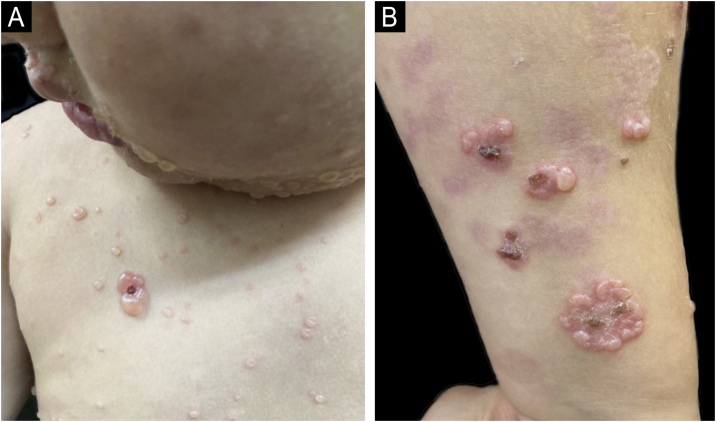
(A) Vesicles with a “string of pearls” pattern on the face, neck, and trunk. (B) Lesions are grouped in rosette-shaped clusters on the forearm.

Histopathology of the skin revealed subepidermal blisters with neutrophilic infiltration in the superficial dermis (Fig. 2A). Direct Immunofluorescence (DIF) showed moderate linear IgA deposition along the Basement Membrane Zone (BMZ) (Fig. 2B). Indirect Immunofluorescence (IIF) and salt-split skin showed negative results. These findings confirmed the diagnosis of LABD.

**Fig. 2:**
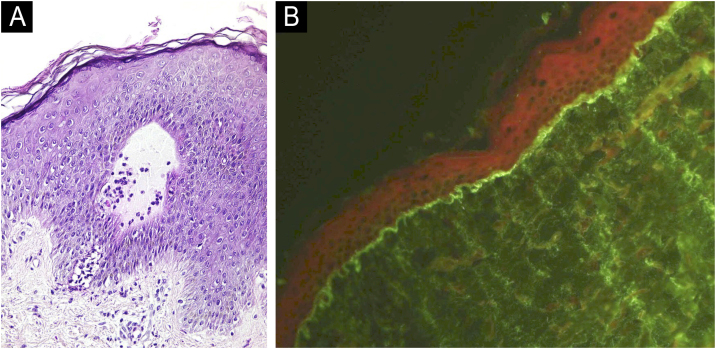
(A) Histopathology of lesion showing subepidermal cleavage with neutrophilic infiltrate surrounding a dermal papilla (Hematoxylin & eosin, ×200). (B) Direct immunofluorescence reveals linear, moderate IgA deposits at the BMZ (×200).

The initial treatment consisted of prednisolone (1 mg/kg/d) and dapsone (2 mg/kg/d) after confirmation of normal glucose 6-phosphate dehydrogenase (G6PD) activity. The patient achieved partial remission for 8-months. He then sought medical assistance after developing an acute headache, dizziness and dyspnea, due to methemoglobinemia. This event led to dapsone withdrawal and the introduction of mycophenolate mofetil (40 mg/kg/d), in association with prednisolone (0.7 mg/kg/d), which were maintained for 10-months, maintaining partial remission. During this period, he developed important side effects, such as Cushing syndrome, obesity (49.5 kg; BMI 35.24 kg/m^2^; P99 for age), and bacterial and herpes simplex infections, treated with cephalosporins and valacyclovir.

After failing to achieve complete remission for 2-years, the patient received two doses of intravenous rituximab (1 g with a 2-week interval, adult weight was considered), with prednisolone (0.5 mg/kg/d), attaining complete remission afterwards (Fig. 3) with no adverse effects. After 8-months, the disease relapsed, and the patient was submitted to a second cycle of rituximab (1 g with a 2-week interval). No adverse effects or relapse occurred in the 6-month follow-up until the present day (Fig. 4).

**Fig. 3:**
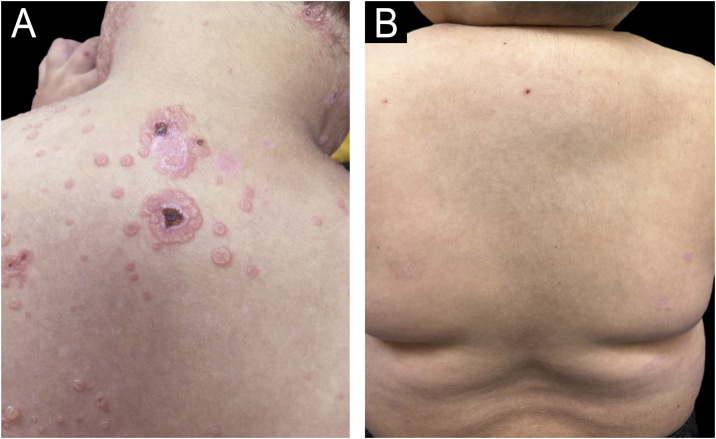
(A) Lesions grouped in an annular “string of pearls” pattern on the back and neck. (B) Residual lesions following the first cycle of rituximab.

**Fig. 4:**
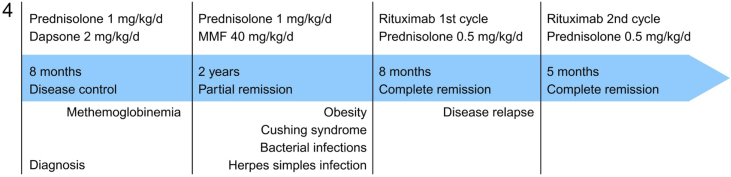
Timeline describing treatment regimen, response to treatment, and adverse effects over the 45-month period following diagnosis.

Adverse effects may impact drug survival in the treatment of AIBD. Adverse events to dapsone include dose-dependent reactions such as hemolysis, methemoglobinemia, and neuropathy, and idiosyncratic reactions such as agranulocytosis and dapsone hypersensitivity syndrome. Adverse effects of prolonged use of systemic corticosteroids include obesity, hypertension, and osteoporosis.^2^

In this context, biologic agents have been described as adjunctive to standard immunosuppressants in AIBD. To date, there are seven reported cases of recalcitrant LABD treated with adjunctive rituximab infusions,^3^, ^4^, ^5^, ^6^, ^7^, ^8^, ^9^ including one pediatric patient^3^ and one with ocular involvement.^4^

The terminology of IgA-dominant subepidermal AIBD and causes for refractory disease are currently a topic of debate. Distinguishing between the BP-230-targeting LABD and the collagen VII-targeting IgA-dominant epidermolysis bullosa acquisita currently relies on the description of the serration pattern of immunoreactants in DIF microscopy and deposition pattern of immunoreactants in IIF on salt-split skin, which do not always yield positive results.^10^ This distinction might prove relevant in future studies concerning IgA-dominant subepidermal AIBD.

There are sparse reports of rituximab in the treatment of recalcitrant LABD. Future studies concerning the role of rituximab and optimization of associated immunosuppressive agents may yield promising results.

## ORCID ID

Pedro Sciammarella Wakisaka: 0009-0002-8012-0258

Larissa Maria Cardoso Lima Rodrigues: 0000-0003-2781-2298

Denise Miyamoto: 0000-0002-4133-4475

Celina W. Maruta: 0000-0002-0541-5526

Claudia Giuli Santi: 0000-0003-3650-4254

Valeria Aoki: 0000-0003-4256-4413

## Financial support

None declared.

## Authors' contributions

Pedro Sciammarella Wakisaka: Writing of the manuscript or critical review of important intellectual content; critical review of the literature; final approval of the final version of the manuscript.

Larissa Maria Cardoso Lima Rodrigues: Intellectual participation in the propaedeutic and/or therapeutic conduct of the studied cases.

Denise Miyamoto: Intellectual participation in the propaedeutic and/or therapeutic conduct of the studied cases.

Celina W. Maruta: Effective participation in the research guidance; writing of the manuscript or critical review of important intellectual content; intellectual participation in the propaedeutic and/or therapeutic conduct of the studied cases.

Claudia Giuli Santi: Intellectual participation in the propaedeutic and/or therapeutic conduct of the studied cases.

Valeria Aoki: Effective participation in the research guidance; writing of the manuscript or critical review of important intellectual content; intellectual participation in the propaedeutic and/or therapeutic conduct of the studied cases; approval of the final version of the manuscript.

## Research data availability

Does not apply.

## Conflicts of interest

None declared.

## References

[1] Caux F., Patsatsi A., Karakioulaki M., Antiga E., Baselga E., Borradori L. (2024). S2k guidelines on diagnosis and treatment of linear IgA dermatosis initiated by the european academy of dermatology and venereology. J Eur Acad Dermatol Venereol..

[2] Bystryn J.C., Steinman N.M. (1996). The adjuvant therapy of pemphigus. An update. Arch of Dermatol..

[3] Mitra D., Bhatnagar A., Singh G., Sandhu S. (2022). Successful treatment of refractory chronic bullous disease of childhood with rituximab. Indian Dermatol Online J..

[4] Steger B., Madhusudan S., Kaye S.B., Stylianides A., Romano V., Maqsood S.E. (2016). Combined use of rituximab and intravenous immunoglobulin for severe autoimmune cicatricial conjunctivitis-an interventional case series. Cornea..

[5] Lamberts A., Euverman H.I., Terra J.B., Jonkman M.F., Horvath B. (2018). Effectiveness and safety of rituximab in recalcitrant pemphigoid diseases. Front Immunol..

[6] Dhillon R., Park L., Gabros S., Nguyen T., Skopit S. (2022). Rituximab for linear immunoglobulin A bullous dermatosis. Dermatol Reports..

[7] Nedosekin D., Wilson K.D., Campbell K., Shalin S., Wong H.K. (2021). Immunologic overlap in a case of linear IgG/IgA bullous dermatosis responsive to rituximab. JAAD Case Rep..

[8] İslamoğlu Z.G.K., Akyürek F.T. (2019). A case of recalcitrant linear IgA bullous dermatosis: successfully treated with rituximab. Dermatol. Ther..

[9] Pinard C., Hebert V., Lecuyer M., Sacre L., Joly P. (2019). Linear IgA bullous dermatosis treated with rituximab. JAAD Case Rep..

[10] Van Beek N., Holtsche M.M., Atefi I., Olbrich H., Schmitz M.J., Pruessmann J. (2024). State-of-the-art diagnosis of autoimmune blistering diseases. Front Immunol..

